# Current practices and challenges of outpatient parenteral antimicrobial therapy: a narrative review

**DOI:** 10.1093/jac/dkae177

**Published:** 2024-06-06

**Authors:** Zenaw T Wolie, Jason A Roberts, Mark Gilchrist, Kate McCarthy, Fekade B Sime

**Affiliations:** UQ Centre for Clinical Research, Faculty of Medicine, University of Queensland, Brisbane, QLD 4029, Australia; UQ Centre for Clinical Research, Faculty of Medicine, University of Queensland, Brisbane, QLD 4029, Australia; Herston Infectious Diseases Institute (HeIDI), Metro North Health, Brisbane, Queensland, Australia; Departments of Pharmacy and Intensive Care Medicine, Royal Brisbane and Women’s Hospital, Brisbane, QLD 4029, Australia; Division of Anaesthesiology Critical Care Emergency and Pain Medicine, Nîmes University Hospital, University of Montpellier, 30029 Nîmes, France; Department of Pharmacy/Infection, Imperial College Healthcare NHS Trust, London, UK; Department of Infectious Diseases, Imperial College, London, UK; Royal Brisbane Clinical School, Faculty of Medicine, The University of Queensland, Brisbane, Queensland, Australia; Department of Infectious Diseases, Royal Brisbane and Women’s Hospital, Brisbane, Queensland, Australia; UQ Centre for Clinical Research, Faculty of Medicine, University of Queensland, Brisbane, QLD 4029, Australia

## Abstract

Extended hospitalization for infection management increases inpatient care costs and the risk of healthcare-associated adverse events, including infections. The growing global demand for healthcare, the diminishing availability of hospital beds and an increasing patient preference for care within their own home have been the primary drivers of the expansion of hospital-in-the-home programmes. Such programmes include the use of IV antimicrobials in outpatient settings, known as outpatient parenteral antimicrobial therapy (OPAT). However, OPAT practices vary globally. This review article aims to describe the current OPAT practices and challenges worldwide. OPAT practice begins with patient evaluation and selection using eligibility criteria, which requires collaboration between the interdisciplinary OPAT team, patients and caregivers. Depending on care requirements, eligible patients may be enrolled to various models of care, receiving medication by healthcare professionals at outpatient infusion centres, hospital clinics, home visits or through self-administration. OPAT can be used for the management of many infections where an effective oral treatment option is lacking. Various classes of parenteral antimicrobials, including β-lactams, aminoglycosides, glycopeptides, fluoroquinolones and antifungals such as echinocandins, are used globally in OPAT practice. Despite its benefits, OPAT has numerous challenges, including complications from medication administration devices, antimicrobial side effects, monitoring requirements, antimicrobial instability, patient non-adherence, patient OPAT rejection, and challenges related to OPAT team structure and administration, all of which impact its outcome. A negative outcome could include unplanned hospital readmission. Future research should focus on mitigating these challenges to enable optimization of the OPAT service and thereby maximize the documented benefits for the healthcare system, patients and healthcare providers.

## Introduction

Infections remain a leading cause of patient morbidity and mortality globally.^1^ Severe infections often require prolonged periods of hospitalization for clinical management, including treatment with parenteral antimicrobials.^2^ However, prolonged hospital stays put a huge burden on the healthcare (hospital) system and the patients, causing an increased risk of healthcare-acquired infections (HCAIs), bed availability shortages, work or school absences, and social isolation.^2–4^ Traditionally, stable patients who would otherwise be discharged from the hospital would remain hospitalized for completion of the IV antimicrobial course when a switch to oral (PO) therapy is not possible. In such patients, the use of a butterfly scalp vein set for IV access was common and facilitated frequent IV administration without limiting patients’ mobility within the hospital.^5^ This historical experience led to the development of outpatient parenteral antimicrobial therapy (OPAT),^6^ a subcomponent of what is known as hospital-in-the-home (HITH)^7^ or hospital-at-home (HaH)^8^ programmes, depending on the jurisdiction.

OPAT is practised differently across the world due to several factors, including the structure of local health delivery systems, geography, availability of specialist services, availability of wound care or other support services, cultural factors, and the diverse socioeconomic needs of patients influencing their preferences.^9^ The impact of these differences in practice on the outcomes of OPAT service appears largely unstudied, as most of the existing reports on safety, efficacy and cost-effectiveness are based on the results of studies from either a single health system/centre or a specific geographical area. To date, comprehensive data showing current global OPAT practices and operational challenges across different settings of practice are not available. Therefore, this article aims to review the published literature and describe the current practices and challenges of OPAT.

## Current global OPAT practices

### OPAT structure and team composition

**Figure 1.**
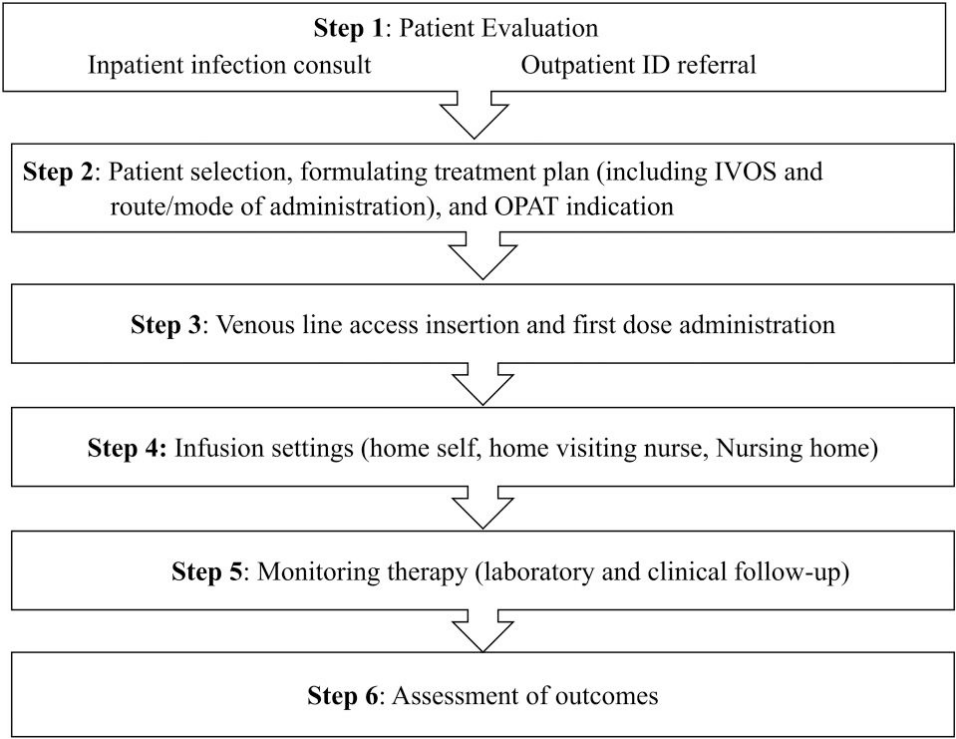
Structure of the OPAT programme. ID, infectious disease; IVOS, intravenous to oral switch; OPAT, outpatient parenteral antimicrobial therapy.

The OPAT structure is organized hierarchically; first, the patient must be evaluated at the referral point before being sent to the OPAT unit where the treatment is given.^10^ At this stage, the patient is seen by the OPAT team at the referral hospital, or the patient is referred to the clinic by the community provider.^11^ If the patient is deemed appropriate for the programme, the logistics and other sequential activities are arranged to enrol the patient (Figure 1).^11^

OPAT programme organization and operations vary across settings and are adapted to local requirements/healthcare structures.^12^ In all operational settings, effective OPAT delivery services require a multidisciplinary team integrated with the patients and/or caregivers.^13^ Although each healthcare setting may have a unique OPAT team composition, it is recommended that the team, at a minimum, should consist of a physician, a clinical pharmacist with antimicrobial expertise, and an OPAT nurse specialist.^13–15^ In many cases, administration staff knowledgeable about OPAT are also included.^13^ The responsibilities of each team member are clearly defined in terms of managerial and clinical roles.^16^ The success of OPAT practice in various OPAT modes of care depends on effective coordination and communication within such a multidisciplinary team.^17–19^

### OPAT models of care

A model of care broadly describes the manner in which health services are provided. Within OPAT, three different models of care (administration of antimicrobials) are available.^15,20–24^ Antimicrobials are administered either by (i) healthcare professionals via an outpatient infusion centre or attending a hospital clinic, (ii) by healthcare professionals visiting patients at home, or (iii) self-administration by appropriately trained, competent, and adherent patients or family members.

In community- or hospital-based outpatient infusion centres, patients attend regular appointments for medication administration.^25^ Some of the advantages of this model include regular monitoring through in-person visits, pharmacy support for patient education, and reduced nursing travel costs. However, it has some disadvantages, including reliable patient transportation requirements and limited drug options owing to the need for once-daily administration.^26,27^ This model is potentially suitable for patients who are unable or unwilling to infuse medication at home and would prefer to have some greater control over the timing of care.^26^

A home-based model of care is more convenient for most patients, particularly those with mobility limitations requiring an ambulance service to travel to the infusion centre.^28^ One component of this model is where health professionals (mostly nurses)^24^ administer antimicrobials at patients’ homes, with daily visits for optimal care.^29^ This model of care ensures safe therapy completion, enables patients to recover in a friendly environment, and offers the opportunity for home inspection and supervised administration. However, it can be challenging where community nursing is not always available. In addition, the travel cost for nurses, including their time, can be a significant limitation.^30^ Staff safety risk also must be managed with regard to pets, other people living in the home, and occasionally the patients themselves.

An alternative home-based model of care is the self-administration OPAT model of care (sOPAT). In this model of care, patients, caregivers or family members receive adequate training to self-administer antimicrobials in aseptic condition, thereby significantly reducing the need for home visits by nursing staff.^26^ Self-connecting devices used in this setting are patient-friendly and are different from the connector sets used by the nursing staff. In certain OPAT settings, a nursing home visit is scheduled on discharge day and afterwards as needed.^26^ In some settings, patients are instructed to visit the emergency department and given a contact number for use if any problem occurs after hours or on the weekends.^31,32^ With proper handling of antimicrobials and careful patient selection, sOPAT programmes have been demonstrated to be a safe, effective and cost-saving approach.^33–35^ Particularly, the use of elastomeric infusion devices enhances OPAT feasibility with continuous 24 h antibiotic infusion. This approach also avoids the need for multiple daily visits from healthcare practitioners for therapies requiring multiple daily doses.^28,36–38^ Even twice-daily self-administration is feasible with such self-connecting devices for OPAT, which is clinically more attractive by enabling the use of multiple daily regimens as needed.^33,39^ In some jurisdictions, such as the USA, administering medication three times per day with these devices may also be an option, for example, in patients with mycobacterial infections requiring imipenem/cilastatin.

However, patients who require additional nursing care or lack home infusion insurance benefits are typically enrolled in another OPAT model of care known as a skilled nursing facility (SNF). In SNFs, on-site nurses handle all infusion tasks, along with other activities like physical therapy or wound care.^26^ Because an SNF is a healthcare facility, patients are more likely to encounter resistant organisms.^40^ Overall, this option incurs significantly higher costs to the healthcare system compared with other OPAT models but may reduce the patient’s out-of-pocket expenses.^26^ Nonetheless, there are multiple challenges with this and each of the other models of care in terms of their operational and organizational structures.^24^

### Patient eligibility criteria for the OPAT programme

Not all patients with a clinical condition requiring antimicrobial therapy may be eligible for the OPAT programme. OPAT enrolment should be based on patient evaluation and selection, aiming to maximize favourable treatment outcomes and reduce treatment failure and subsequent complications.^11,25,41,42^ It is important to recognize that the criteria used by OPAT practitioners—encompassing clinical, patient or model-related factors—are not absolute but rather relative criteria to aid in determining eligibility (Table 1).^25^ OPAT-eligible patients should be clinically stable, respond well to current therapy, and should not have other active medical conditions that require hospitalization that cannot be managed safely by other specialists on the team. Prior to enrolment, the OPAT team should confirm that parenteral therapy is actually needed, and no oral alternative agent is available. Additional criteria, including diagnosis, availability of medication-administration line and appropriate antimicrobials, patients’ consent and their active involvement, the presence of family members if required, accessibility and affordability of infrastructure (e.g. transportation, infusion centres), and safety and suitability of the home environment, including nursing safety to visit the home, should be considered.^43–47^ It is essential to underline that these criteria are comparative considerations, and the choice to proceed with OPAT should be carefully balanced against the drawbacks of extended hospital stays. Ultimately, a mutual agreement between the patient and the OPAT team, together with a definitive clinical management plan, is essential.^27^

**Table 1.** The OPAT programme's eligibility requirements for patients

Clinical criteria	Patient-related criteria	Model of care–related criteria
The patient must be clinically stable Oral or nasogastric feeding is possible Absence of comorbidity with an intrinsic indication of hospital admission Availability of suitable antibiotic therapy Superior IV therapy effectiveness than oral Diagnosis should be certain No acute oxygen requirement Appropriate line access for antibacterial administration Access to TDM, if required No adverse drug reaction that prohibited therapy	Consent to OPAT and understand the provision of care that will occur with this service The patient should actively collaborate and adhere to treatment Able to care for oneself or the infant Risk of non-adherence in PWID Access to telephones at home	Access to clinics or infusion centres if required Access to transportation, if required Access and hygiene condition of the home Access to a refrigerator, electricity and running water Safety for nursing staff if visiting home is required Acceptable environmental temperature range Availability of a 24 h access line

OPAT, outpatient parenteral antimicrobial therapy; PWID, people who inject drugs; TDM, therapeutic drug monitoring.

### OPAT in special populations

Although an OPAT programme offers high-value care, it may be challenging for people who inject drugs (PWID), undomiciled individuals, and other special populations.^48^ Ashraf *et al.*^45^ described that one-third (total = 129) of PWID who were discharged to SNFs failed to complete the planned parenteral therapy. However, despite limitations such as loss of follow-up and a relatively higher rate of complications compared with non-drug users, OPAT can be feasible and safe for undomiciled individuals and PWID with serious infections, provided there is careful patient selection, good patient engagement, and adequate resource allocation.^47–49^ For instance, PWID with non-acute bacterial skin and skin structure infections treated with dalbavancin have shown a 71% success rate,^50^ comparable to cure or improvement rates in non-PWID (ranging from 61.1% to 100%).^51^ For a comprehensive review of the safety and efficacy of OPAT in PWID, the reader is referred to the article by Suzuki *et al*.^52^

Additionally, limited data suggest OPAT's safety in nonagenarians, clinically stable neonates, and young infants. In a study by Shrestha *et al*.,^44^ no significant increase in OPAT-related complications was observed for nonagenarians compared with younger patients. The feasibility and effectiveness in neonates/young infants was demonstrated in a study from the Royal Children’s Hospital Melbourne, reporting a high success rate of treatment completion (100% in neonates and 96% in older infants) and low rates of unplanned readmissions.^43^ Similarly, a study on young infants under 3 months old receiving OPAT in Salt Lake City, Utah, reported that the treatment complication rate was comparable to that in older children, with no treatment failures or disease progression.^53^ These findings suggest that with careful patient selection and monitoring, OPAT can be a viable option for providing essential antimicrobial therapy to patients at the extreme ends of the age spectrum.

### Frequently used antimicrobials in OPAT practice

A wide range of antimicrobials have been used in the OPAT practice, including β-lactams, aminoglycosides, glycopeptides, tetracyclines, lipopeptides, and, in some cases, fluoroquinolones (e.g., levofloxacin and ciprofloxacin) and antifungals.^20,21,54–58^ Due to geographical differences in patient conditions, severity of disease, available models of care, and licensing of agents, the patterns of antimicrobial use across countries are divergent. Selecting the right antimicrobial for a particular infection is dependent on local requirements and guidelines.

#### Antibacterial agents

##### β-Lactam antibiotics.

**Figure 2.**
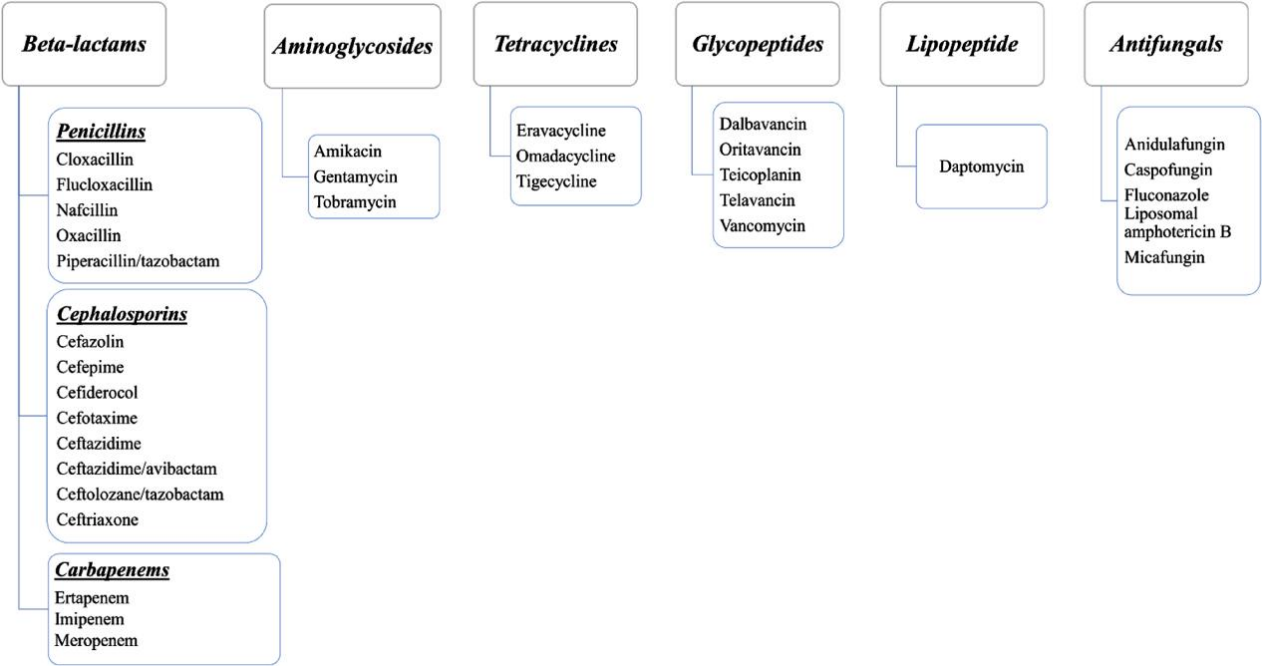
Antimicrobials that are used in OPAT settings across countries.

In OPAT settings, IV β-lactam antibiotics (including penicillins and cephalosporins) (Figure 2) play a crucial role in treating bacterial infections, ranging from moderate to life-threatening.^21,45,59–63^ Carbapenems, particularly meropenem, have gained interest despite challenges related to stability during continuous 24 h infusions.^64^ Additionally, once-daily ertapenem, with its extended elimination half-life and broad-spectrum activity, is increasingly used in OPAT.^65^

β-Lactam antibiotics exhibit time-dependent killing activity, where maintaining the free drug concentration above the MIC is critical for bactericidal effects.^59^ However, the short elimination half-life and post-antibiotic effects of most β-lactams often necessitate multiple daily doses with standard intermittent infusion regimens. Unfortunately, this approach is impractical in OPAT due to increased nursing visit requirements and infusion line complications. Therefore, β-lactams that require less frequent dosing (ideally once daily or less) are chosen, or those with short half-lives are often administered via continuous infusions (CIs) over prolonged periods. For β-lactams (with some exceptions such as ceftriaxone and ertapenem, which are typically not given as CI), CI of the total daily dose, administered once or twice daily, can conveniently optimize their exposure and is proven safe and effective in clinical practice.^59,66,67^ For instance, a cohort study found that CI of certain β-lactams improved pharmacodynamic target concentrations compared with a standard bolus infusion.^37,59^

However, the poor stability of some β-lactams limits the use of CI in OPAT settings where the environmental temperatures can be high (≥32 °C). Active cooling and therapeutic drug monitoring (TDM) can help mitigate stability issues. Alternatively, co-administering probenecid with β-lactams may prolong their half-lives and potentially eliminate the need for CI.^68^ Nevertheless, clinical data on most β-lactams in this context remain limited.

##### Aminoglycosides.

In outpatient settings, aminoglycosides remain clinically important antimicrobials for treating various infections, including aerobic Gram-negative and Gram-positive infections, mycobacterial infections, urinary tract infections (UTI) and enterococcal endocarditis. Commonly used aminoglycosides in OPAT include amikacin, gentamicin, and tobramycin (Figure 2).^58,63,69,70^ For instance, amikacin is used for treating ESBL-producing *Escherichia coli* (ESBL-EC) infections with close toxicity monitoring.^71^

Aminoglycosides exhibit concentration-dependent bactericidal activity, with optimal efficacy achieved through once-daily bolus infusion with a reduced occurrence of drug-induced toxicity. With appropriate monitoring, the dosing regimen of aminoglycosides is well suited to the OPAT programme,^72^ with no concerns of drug instability given their short bolus infusion.

##### Glycopeptides.

In OPAT, vancomycin, the oldest in its class, treats Gram-positive bacterial infections, including MRSA.^73,74^ It is used for conditions like periprosthetic joint infections, osteomyelitis, complicated skin and soft tissue infections (SSTIs), and endocarditis, which often require treatment durations of several weeks to months.^75^ Treatment with vancomycin via CI using elastomeric pumps shows safety and efficacy, with up to a 100% cure rate.^37,75^ These findings, along with no significant difference in clinical effectiveness between CI and an intermittent infusion of vancomycin, make it a viable option in OPAT.^76,77^ Vancomycin is widely used due to its affordability.^78^

Teicoplanin, a semi-synthetic form of vancomycin, is another promising option for OPAT.^79^ It exhibits activity against *vanB* vancomycin-resistant enterococci and has fewer reported adverse effects than vancomycin. It is used to treat acute and life-threatening infections caused by β-lactamase-producing Gram-positive pathogens.^80^ Teicoplanin has demonstrated effectiveness in treating SSTIs in OPAT settings.^79^ Teicoplanin’s convenient pharmacokinetic properties contribute to its widespread use in OPAT.^81^ With a half-life ranging from 48 to 182 h in renally non-compromised patients, it can be administered once daily or even three times weekly without compromising efficacy,^80^ which supports its use in OPAT practice. Teicoplanin offers a broader therapeutic index and comparable efficacy to vancomycin, but careful administration (avoiding shaking to prevent foam formation) is essential.^80^ Regular TDM may be necessary due to significant pharmacokinetic variability.^82^

Telavancin, a once-daily-administered antibiotic, is authorized in the USA for treating complicated skin and skin structure infections (cSSSIs) in adults due to its suitable pharmacokinetic properties that facilitate the practice of OPAT.^83–85^ When treatment options are lacking, it is also approved for managing hospital-acquired and ventilator-associated bacterial pneumonia (HABP and VABP) caused by susceptible *Staphylococcus aureus* isolates.^86^ In Europe and Canada, telavancin controls nosocomial pneumonia caused by MRSA and treats HABP/VABP and cSSSIs due to susceptible Gram-positive pathogens.^87^ Additionally, dalbavancin and oritavancin, both long-acting lipoglycopeptides, significantly reduce dosing frequency (where a once-weekly or even a single dose is sufficient).^85^ Their parenteral forms are preferred alternatives for OPAT patients because their administration does not require prolonged vascular access and hence increases patient acceptability.^88^ These agents, approved for treating acute bacterial SSSIs, may also be useful off-label for bloodstream infections, infective endocarditis (IE), and bone and joint infections, including prosthetic joint infections and osteomyelitis.^85,89–93^ In OPAT, IV oritavancin and dalbavancin can be helpful for complicated infections in special patient populations, such as PWID and the undomiciled.^94^

##### Tetracyclines.

Parenteral use of certain tetracyclines in OPAT presents an invaluable option for treating infections outside the hospital setting. Eravacycline, a novel fluorocycline antibiotic, exhibits broad-spectrum activity against both Gram-positive and Gram-negative pathogens, making it a promising candidate for OPAT in cases where multiple organisms are suspected.^95^ It is approved for twice-daily dosing without adjustment for renal dysfunction.^95^ Tigecycline, which belongs to the glycylcycline class, offers activity against MDR bacteria, including MRSA and ESBL-producing Enterobacterales, making it a valuable option in settings where resistance patterns are complex.^96^ Although licensed for twice-daily IV administration, its long half-life allows for once-daily dosing in OPAT settings.^97^ Omadacycline also demonstrates broad-spectrum activity against various pathogens, including Gram-positive, Gram-negative and atypical bacteria, and may be considered in OPAT.^98^ Therefore, these antibiotics, with distinct mechanisms and activity spectra, offer clinicians options for tailored therapy in outpatient settings, facilitating effective management of diverse infections while minimizing hospitalization duration and associated costs. However, careful patient selection, monitoring, and adherence to antimicrobial stewardship may be vital to optimize outcomes and counter resistance and adverse effects.

##### Lipopeptides.

Daptomycin is a bactericidal lipopeptide used in OPAT for osteomyelitis, joint infections, uncomplicated SSSIs and cSSSIs, and IE due to Gram-positive pathogens.^99–101^ It has a longer duration of action (a half-life of about 8 h) with a post-antibiotic effect of nearly 7 h.^102^ A once-daily administration, a short infusion time (2 min bolus infusion), and a good safety profile that does not necessitate routine TDM make daptomycin an attractive option in OPAT.^99^ Clinical studies have demonstrated its efficacy and safety, with up to 60% fewer adverse drug events compared with drugs like vancomycin.^102,103^

##### Fluoroquinolones.

Quinolones like ciprofloxacin and levofloxacin exhibit excellent oral bioavailability (85%–100%), comparable to their parenteral administration.^78^ The ratio of the area under the plasma concentration curve to MIC and the peak/MIC ratio are the common exposure metrics for quinolones, which are often optimized by intermittent infusion regimens, unlike some time-dependent antibiotics, such as β-lactams, which may require prolonged infusion.^104^ In OPAT, albeit to a limited extent, IV fluoroquinolones are used alongside β-lactam and non-β-lactam antibiotics when oral dosing is challenging due to impaired absorption.^78^ Valinetz *et al*.^21^ found that parenteral levofloxacin/ciprofloxacin managed 8% (*N =* 50) of adult participants in orthopaedic hardware-related infection outcomes. In a Spanish hospitals-in-the-home programme, levofloxacin and ciprofloxacin constituted only 3.5% and 0.82% of the total prescribed antimicrobials (*N =* 5004) for treating bacterial infections, respectively.^105^ Additionally, Theocharis *et al*.^106^ reported that 5.4% and 4.3% of 91 patients received levofloxacin and ciprofloxacin monotherapy in OPAT, respectively.

#### Antifungal agents

In OPAT programmes, antifungal agents are used infrequently due to their systemic toxicities and high unplanned readmission rates.^107–110^ Oral triazoles are preferred for treating fungal infections, but resistance [e.g., triazole resistance by *Aspergillus fumigatus* (10% incidence) and the increasing resistance in *Candida* species], toxicity, and drug interactions present challenges.^109,111^ Recent experience indicates that, with proper patient selection and monitoring, antifungals can be safely used in OPAT.^107,108,110–112^

Common antifungal agents used in OPAT are depicted in Figure 2. Amphotericin B is the oldest parenteral antifungal drug that has been used in OPAT, and it is routinely indicated for many invasive fungal infections.^110^ A study from the USA and Ireland indicated that 47% and 14% of physicians, respectively, reported experiences of using amphotericin B in OPAT.^109^ Amphotericin B, especially its liposomal form, is a well-established OPAT antifungal with fewer side effects and a dosing regimen of every 72 h, leveraging its long half-life of up to 152 h.^107,111^ This makes it a convenient option for OPAT, ensuring sustained therapeutic levels with less frequent administration.^111^

Fluconazole, with equal bioavailability (90%) for oral and IV forms, is another option for OPAT, particularly when oral administration is problematic.^78^ It has been used for a range of infections, including those in haematology and oncology patients, gastrointestinal and genitourinary infections, endocarditis, CNS infections, upper respiratory, and UTIs.^113^

Echinocandins like micafungin have also been used in OPAT, showing good cure rates for chronic pulmonary aspergillosis with minimal side effects.^112^ Caspofungin and anidulafungin are potential OPAT candidates, though data on their use are limited.^109^ In Spain, caspofungin was the most frequently prescribed antifungal prescription in home-based IV therapy, accounting for 36.5% of the total prescriptions (*N =* 63), followed by parenteral fluconazole (31.75%) and liposomal amphotericin B (12.7%).^105^

#### Other antimicrobials

In OPAT, parenteral forms of metronidazole, clindamycin, linezolid, and co-trimoxazole have been occasionally used despite their high oral bioavailability (≥85%) when there are practical issues or financial constraints limiting their oral use.^78,114,115^ Parenteral metronidazole, often combined with other antibiotics (e.g., with ceftriaxone, cefazolin and meropenem), treats various infections, including dental,^116^*Clostridioides difficile* colitis,^117^ gastrointestinal/genitourinary,^113^ CNS,^113^ intra-abdominal, joint and bone,^58,105,118^ and infections due to *Bacteroides fragilis*, *Enterococcus faecalis* and *Bacteroides caccae.*^119^

In some settings, linezolid and clindamycin have been used parenterally in OPAT for orthopaedic infections, skin infections, and IE when oral administration is not feasible.^115,120,121^ Tedizolid, a novel oxazolidinone antimicrobial known for its convenient once-daily dosing schedule and supposedly lower toxicity, is increasingly used in Europe and the USA for the treatment of acute bacterial skin infections.^120,122^ These attributes of tedizolid contribute to improved patient compliance and satisfaction, and overall quality of care in the OPAT context.

In OPAT, the use of IV antivirals like aciclovir and ganciclovir is considered feasible, although there is a scarcity of data on their safety and effectiveness.^57,69,105,123,124^ IV ganciclovir is mainly used to prevent and treat infections caused by cytomegalovirus-related infections, including hepatitis, retinitis, and pneumonitis.^125^ Similarly, parenteral aciclovir is administered to manage infections from herpes simplex virus, varicella-zoster virus in the CNS, and other widespread viral conditions in OPAT.^11^

### Antimicrobial stewardship and OPAT

Generally, antimicrobial stewardship (AMS) focuses on choosing the most effective, safe, and narrow-spectrum agents with minimal adverse effects to optimize outcomes, reduce antibiotic costs, and preserve future treatment options.^12^ In practice, this principle of AMS presents a challenge to the OPAT programme. In OPAT, the need for convenience in dosing administration to enhance early discharge or avoid admission may be superior to an agent’s spectrum of activity.^126^ Therefore, OPAT services need to balance these priorities and the AMS preference for using narrow-spectrum agents. However, this decision is challenged by multiple factors, including the limited availability of long-acting narrow-spectrum antimicrobials, insufficiently validated stability data for some narrow-spectrum agents, or the need for frequent TDM of some narrow-spectrum agents, leading to the use of broad-spectrum antimicrobials with suitable characteristics for OPAT.^18,126^

In recent years, a switch from IV to oral antimicrobial therapy has been increasingly considered within HITH/OPAT programmes.^127^ Evidence suggests that oral antimicrobials may be non-inferior to IV therapy for certain infections that were traditionally considered to require IV therapy. Trials like Oral Versus Intravenous Antibiotic Treatment (OVIVA) and partial oral therapy with IV therapy (POET) have demonstrated that oral therapy is non-inferior to IV therapy in terms of outcomes and safety for conditions such as bone and joint infections and IE.^127,128^ Thus, the perception that IV antimicrobials are always superior to oral antimicrobials is changing, such that the use of oral agents for severe infections has been a growing area of practice. The shift from IV to oral has led to the concept of complex outpatient antimicrobial therapy (COpAT), which aligns with the broader goals of AMS, including minimizing the risk of antibiotic resistance and optimizing the use of antimicrobial agents.^129^ In the OPAT/COpAT setting, AMS strategies may involve transitioning to COpAT from traditional OPAT, employing the shortest effective duration of antimicrobial therapy, opting for agents with less frequent dosing, and implementing regular laboratory and safety monitoring.^88^

### Antimicrobial-administering devices used in OPAT practice

The success of an OPAT service is reliant on the proper use of antimicrobial administration devices such as vascular access devices and drug infusion devices.

#### Vascular access devices

Vascular access devices (VADs) are devices inserted into central or peripheral veins or implanted under the skin, enabling antimicrobial administration, including via the subcutaneous route. There are different options of VADs for use in OPAT, each with their own attributes.^130–133^ Broadly, they are categorized into central and peripheral VADs. The characteristics of each device, including their length of use, indication, technique of insertion and relative advantages, are summarized in Table 2.

**Table 2.** Summary of types of VADs used in OPAT and their descriptions

VAD type	Place of insertion	Duration of use	Advantages	Disadvantages
PICC	Central	Short-term but have been used for longer when required	Easy to place and remove by a specially trained registered nurse	Potential for occlusion Risk of DVT Risk of CRBSI
PVC	Peripheral	Short-term (less than 6 d)	Easy to place and remove Rare serious complications	Short dwell time Discomfort due to frequent replacements Frequent non-serious complications Unsuitable for longer treatment time Risk of extravasation Risk of CRBSI
Midline	Peripheral	Usually short to intermediate (6–14 d)	Easy to place and remove Cheaper than PICC	Frequent non-serious risk of DVT and CRBSI
Non-tunnelled CVCs	Central	Days to weeks	Percutaneous insertion	Account for the majority CRBSIs Require local anaesthesia Not recommended for home use (complications)
CVC (tunnelled)	Central	Months to years	Suitable for longer treatment time Low risk of extravasation Lower rate of infection than non-tunnelled CVCs	Frequent placements destroy the central vessels Difficult to place (surgical insertion) and remove Requires much supervision Increased cost Risk of DVT andCRBSI
Implantable ports	Central	Long-term (months to years)	Cosmetically less noticeable (low visibility of port) Patient comfort	Require surgical insertion Require local or general anaesthesia High cost Risk of subcutaneous pocket infection Risk of CRBSI

CRBSI, catheter-related bloodstream infection; CVC, central vascular catheter; DVT, deep vein thrombosis; OPAT, outpatient parenteral antimicrobial therapy; PICC, peripheral inserted central catheter; PVC, peripheral vascular catheter; VAD, vascular access device.

Peripherally inserted central catheters (PICCs) are the most commonly used VADs in OPAT patients^11,132^ and are suitable to administer medications for longer than 7 days, facilitating early discharge from the hospital.^134^ PICCs are more convenient and safer when inserted at the basilic vein due to their large diameter; however, cephalic and brachial veins can also be considered.^135^ PICCs are preferred for use with CIs of vesicants, including vancomycin, aciclovir, and nafcillin,^57^ and other chemically irritating or non-peripherally compatible solutions.^134^ Safe insertion, cost-effectiveness, and self-care compatibility that facilitate outpatient use are important additional benefits of PICCs. Other central venous access devices, such as implant ports and tunnelled catheters, may be considered for a longer period of treatment (months to years).^60,136^ They are often present due to other medical indications.

Peripheral vascular catheter (PVC) devices may be used in conditions where central venous access is not possible for various reasons and when treatment involves the infusion of peripherally compatible solutions for 5 days or less.^134^ These devices enable access to the veins and arteries of the arm and occasionally to the legs. PVCs are short catheters, and they are safe and cost-effective when used for short periods of time. These types of catheters are not recommended for vesicant therapies.^134^ In addition, PVC replacement should be no more frequent than 72 h, given that the catheter remains functional and shows no signs of inflammation or infection.^137^

Midline catheters (MCs) are a type of PVC device used in OPAT to administer non-vesicant antibiotic therapies. They are long peripheral catheters inserted into the antecubital or upper arm vein.^134^ Infrequent site changes, a low risk of complications (phlebitis) and low rate of infections are among the advantages of MCs over short peripheral catheters, PICCs and other central catheters. Patients using MCs are less likely to get pneumothorax during insertion. Moreover, MCs are cheaper or nearly comparable to PICCs, and therefore MCs are used as alternatives to PICCs in certain circumstances,^133^ for example, when administration of non-irritant IV medication for more than 5 days is required in resource-limited settings. A recent report evaluating their safety suggested that MCs could be safely used for periods not exceeding 14 days;^138^ there were insufficient data on their safety for longer durations.

Depending on their circumstances, OPAT practitioners can choose the appropriate VADs. The choice of suitable administration devices relies on several factors, including medication type, duration and frequency of therapy, patient wishes and age, venous condition, and the risk of bloodstream infections and thrombosis.^10^ The selection of the appropriate VADs considering these factors, as well as identifying the right administration site, is critical to ensuring positive patient outcomes. Further to this, placing the VAD correctly to last for the length of therapy or until the minimal replacement period of time is especially important to reduce complications and treatment failure. In addition, for OPAT self-administrators, effective patient or caregiver training on the devices and administration methods is of paramount importance to ensure safety and medication adherence.^13,123^

#### Infusion devices

In OPAT, VADs may be connected to different infusion devices, including devices for gravity drip, ambulatory electronic infusion devices (EIDs), or elastomeric infusion pumps (EIPs) for easy medication administration.^19,139^

Portable EIDs are power-dependent and use battery power to pump antimicrobials from the reservoir. Although these portable EIDs deliver the medication with a more accurate flow rate, their use is limited by programming errors, pumping noise, amenability for self-connection in sOPAT, and their high cost.^140^

EIPs are disposable devices that apply pressure to infuse medications.^141^ They are power-independent, and the infusion flow rate is produced by the pressure from the balloon reservoir and the resistance from the restrictor element in the infusion line.^142^ Consequently, the patient receives the antimicrobials as the elastomeric pump consistently deflates and gradually pushes solution through the IV tubing. In most home infusion OPAT settings, EIPs are widely used as they are easy to use, cheaper than electronic pumps, independent of gravity, portable, and suitable for active patients to continue their activity while on OPAT.^36,38,139,142^ They play an important role in ensuring continuous and prolonged antimicrobial infusion, improving the quality of life of the patients, improving clinical efficacy, and reducing the overall OPAT cost.^39,143^

Several EIPs are available from different manufacturers with different nominal filling volumes, temperatures, and flow rates. Mostly, their size and flow rate range from 50 to 500 mL and from 0.5 to 250 mL/h, respectively, allowing medications to be infused over 30 min, 1 h, 1.5 h, 12 h, 24 h or more.^139^

The performance of elastomeric pumps is dependent on temperature, and this temperature varies between pumps; most are designed to operate at the intended flow rate when the antimicrobial solution is within a temperature range of 31–34 °C when placed close to the skin.^144^ However, Baxter Intermate infusers are designed to perform at 21.1 °C. In general, OPAT practitioners and patients can expect that for every 1°C above or below this temperature, the flow rate will increase or decrease by approximately 2%–3%.^141,145^ Temperature variation for these elastomeric pumps affects flow rate and may reduce the amount of antimicrobials delivered to the patient.^142^

## Common challenges of OPAT practice

Although OPAT is safe, effective and practical for all age groups, there are a number of challenges that arise during the management of the patient on the service.

### Challenges associated with clinical and laboratory monitoring

Optimal monitoring of OPAT patients is challenging due to logistics, including insufficient financial and/or administrative support and the diversity of locations.^19^ Patients receiving OPAT require scheduled monitoring, which could be either clinical assessment, laboratory testing or both. Such monitoring includes evaluating the response to treatment, ensuring the safety of administered drugs, monitoring line- and infusion device-associated complications, assessing potential drug interactions and managing adverse drug events. However, regular and more frequent monitoring in outpatient settings is more challenging than with inpatient care. Whereas patients receiving OPAT at infusion centres may be monitored daily by the nursing staff, pharmacists or physicians, those treated under the home-based model of care are evaluated less frequently than under the inpatient model of care,^78^ which results in missing laboratory information, limiting assessment of outcomes. In addition, there is variability in monitoring frequency and strategy among OPAT delivery models of care, and the optimal frequency of clinical assessment and laboratory testing is not well established. ^146^ Telemedicine can provide a potential solution enabling wider clinical monitoring of patients in OPAT settings; it has been endorsed by the IDSA and is likely to have a prominent role in future practice.^88,147^

The IDSA and BSAC OPAT guidelines recommend at least a weekly complete blood count with differential, and serum creatinine for all parenteral antimicrobials.^148,149^ For narrow therapeutic index antimicrobials such as aminoglycosides and vancomycin, the guidelines also recommend weekly TDM. Patients taking highly nephrotoxic agents and those with a prescription containing more than one nephrotoxic agent are at high risk of renal complications. Monitoring electrolyte imbalance, drug-induced hepatitis, and cytopenia among patients receiving certain β-lactams (e.g. nafcillin sodium, ceftriaxone, oxacillin and carbapenem), as well as amphotericin B, is recommended.^78^ Daptomycin administration is frequently associated with elevated levels of creatinine phosphokinase, and therefore more frequent (twice weekly) renal function tests and potassium level monitoring are also advisable.^13^

In practice, however, routine monitoring may not be strictly followed, particularly for home-based models of care.^150^ Physician time constraints, distance between the patient’s home and the managing physicians, the need for patients to be seen by multidisciplinary team members, and the challenge of patient mobility are among the barriers to frequent clinical and laboratory monitoring.^78^ Moreover, in some cases, even laboratory results may be lost and unavailable for a physician to intervene due to a lack of effective communication among OPAT practitioners.^151^ Inappropriate clinical and laboratory monitoring, together with unavailability of results, leads to irrational antibiotic dosing, adverse drug reactions, unnecessary therapy prolongation, complications, hospital readmissions and increased healthcare costs.^146^ To effectively address clinical and laboratory monitoring challenges in OPAT, it is crucial to identify strategies for reaching vulnerable patient groups.^13,152^ Integrating advanced technologies into the future of OPAT presents promising solutions. Telemedicine platforms could bridge the distance between patients and healthcare providers, facilitating timely interventions and follow-ups.^88,147^ Mobile applications for real-time data sharing and patient engagement may reduce the incidence of lost laboratory results and support more informed clinical decision-making.

### Antimicrobial-related complications

In outpatient settings, particularly where regular and strict patient follow-up is not practical, the selection of antimicrobials and accurate patient diagnosis are crucial to mitigating medication-related adverse events.^12,19^ Studies have shown that OPAT patients may experience a wide range of antimicrobial-related adverse events (Table 3) due to infrequent follow up compared with inpatient cares.^15,22,163^

**Table 3.** Summary of antimicrobial-related complications encountered in OPAT settings

Antimicrobial	Complications	Incidence	Notes	References
Vancomycin	Adverse drug events such as phlebitis, neutropenia, nephrotoxicity (that leads to AKI, eosinophilia, creatinine changes and electrolyte abnormalities)	High	Independent risk factor for adverse drug events in OPAT May lead to treatment discontinuation and hospital readmission	^15,75,78,117,153,154^
			Risk of renal toxicity increased when vancomycin combined with nephrotoxic agents like fluoroquinolones and piperacillin/tazobactam Has a relatively narrow therapeutic index and requires regular TDM to avoid renal toxicities	^74,146,155^
	Vancomycin infusion reaction	—	Formerly known as ‘red man’s syndrome’, an infusion-related anaphylactoid reaction to vancomycin An infiltrate does not equate to vancomycin infusion syndrome (which is a histamine release–based reaction)	^78,156^
Aminoglycosides	Nephrotoxicity (leading to electrolyte imbalance and AKI) and ototoxicity (which may result in hearing loss), neuromuscular blockage (rarely)	—	Use is uncommon compared with β-lactams, especially in children	^10,108,157,158^
Parenteral β-lactams (oxacillin, ceftriaxone, nafcillin, carbapenems, cephalosporins)	Hypersensitivity, including anaphylactic reactions, thrombocytopenia and eosinophilia	0.5% for anaphylactic reactions	First dose should be given in a setting where staffing and equipment allow appropriate monitoring for anaphylaxis.	^26,78,117,159^
	Transaminitis, rash, acute renal injury, neutropenia	8.4 per 1000 OPAT days for oxacillin	The adjusted odds of an adverse event were 3.3 times greater with oxacillin than cefazolin, ceftriaxone and ertapenem	^153,160^
	Drug-induced hepatitis	Rarely	Ceftriaxone, nafcillin, oxacillin and carbapenems (mostly ertapenem)	^15,78^
	*C. difficile*-associated diarrhoea	15%–20%	Linked to β-lactams and fluoroquinolones, especially third-generation cephalosporins	^11,126^
Vancomycin, ceftriaxone and ertapenem	Antimicrobial infusion reaction including itching, erythema and nausea	6.5%	Although the results showed a 6.5% immediate reaction rate, the authors suggest that monitoring may not be necessary for most patients receiving first-dose IV antimicrobials in outpatient settings, which contradicts the IDSA recommendation	^161^
Ciprofloxacin and levofloxacin	Tendonitis/tendon rupture, cardiac arrhythmias, retinal detachment, peripheral neuropathy	—	—	^26^
Conventional amphotericin B	Nephrotoxicity	—	High association with renal toxicity, worsened with higher doses and prolonged therapy Creatinine levels can rise more than twofold.	^108,111,162^
Liposomal amphotericin B (LAmB)		—	Reduced incidence of nephrotoxicity compared with conventional form, but still considerable	^162^
Micafungin	Abdominal pain, anaemia, fever, headache, electrolyte abnormalities, leukopenia	Up to 16%	—	^112^
Linezolid and tedizolid	Thrombocytopenia, leukopenia, anemia, peripheral neuropathy, and optic neuritis	—	—	^26^

AKI, acute kidney injury; OPAT, outpatient parenteral antimicrobial therapy; TDM, therapeutic drug monitoring.

The variability in antimicrobial-related complications is influenced by diverse patient demographics, disease conditions, socioeconomic aspects and treatment requirements, as well as the pharmacokinetic and pharmacodynamic properties of the antimicrobials. These factors affect treatment duration, frequency and the necessity for regular monitoring. Prolonged treatment, frequent administration of toxic agents, a narrow therapeutic index, potential for harmful drug interactions, urban residence and obesity are linked to higher rates of adverse events.^63,88,113,146^ Immunocompromised individuals and those with comorbidities are particularly at risk.^164^ Adverse drug events tend to be more prevalent shortly after hospital discharge, highlighting the need for vigilant monitoring and regular clinical assessments to minimize toxicity and ensure timely medical interventions.^56,146^

### Device-related complications

Complications from venous access and infusion devices may cause treatment interruptions and hospital readmissions. VAD complications include line-associated infections, vein thrombosis, leaking and line failure, accidental dislodgement, bleeding, occlusion, and insertion site haematoma.^44,58^ Complication severity, magnitude, and frequency vary among devices. The reasons include length of use, patient condition, type and composition of administration devices, and caregiver handling skills.^123^ Line infections are associated with line use duration, female sex and tunnelled central vascular catheter (TCVC) lines.^131^

The frequency and types of device-related complications vary among studies. Shrestha *et al.*^123^ and Keller *et al.*^57^ reported 144 (∼10%) and 43 (12.7%) vascular complications in 1461 and 339 patients, of which 53% and 34 (10%) were due to line occlusion and ocular catheter complications, respectively. A cohort study by Kaul *et al.*^22^ reported that 8.45% of OPAT courses (144 of the total 1704 adult patients) resulted in vascular complications, with line dislodgement or leak complications accounting for 4.40% of all vascular complications. Marsh *et al.* showed that 10% (*N* = 180) of patients experience PICC failure during any treatment course, with 5% of these failures being due to catheter dislodgement.^132^

Vascular complications may vary with catheter types. PICC-associated complications were greater than those associated with Hickman catheters.^123^ However, other studies showed that PICC complications were lower than MCs. According to these reports, PICC-related complications occurred in 37.5%^22^ and 20%^165^ of patients in two separate studies, whereas complications for MCs were reported at higher rates of 62.5%^22^ and 30%^165^ in the same studies, respectively. In contrast, one report suggested that PICCs and MCs share the same safety profile, although further evidence is needed.^166^ PICC complications are fewer compared with tunnelled central venous catheters or implanted vascular ports (port-a-cath).^135^

Antibiotic infusion pumps have limited complications, but lack of an alarm or warning during occlusion, variability of infusion duration, diluent restrictions, and impact on antimicrobial stability when exposed to real-life temperatures are some of the known drawbacks of elastomeric infusers.^36,39^ Programming and user interface errors, pump noise, and higher purchase costs are some of the limitations of EIPs.^140^

### Patient non-adherence and OPAT rejection

Patient non-adherence is a self-directed discharge from OPAT enrolment and a voluntary refusal to follow-up that leads to treatment incompletion. Non-adherence to antimicrobial use impedes OPAT success.^88^ The causes of patient non-adherence during OPAT are multifactorial. In OPAT, younger age, low income, lack of time for medication administration, lack of family support, active drug use, and skipping clinic appointments are some of the factors associated with antimicrobial non-adherence.^167^ Patients may be unable to follow their clinic appointments because of a lack of transportation, being unwell, or being unable to remember the appointment date. Taking multiple antimicrobials, frequent medication administration, and antimicrobial toxicities can also result in non-adherence. Inappropriate laboratory monitoring and OPAT team time constraints, both of which are frequent gaps in OPAT practice, increase the risk of non-adherence.^19^

Management of vulnerable population groups such as PWID, the undomiciled, and patients with an underlying psychiatric illness is challenging due to non-adherence, which causes poor treatment outcomes.^48,167^ Active drug users, patients with substance use disorders, polysubstance users, and undomiciled patients have nearly double the risk of non-adherence.^167^

Non-adherence has multiple consequences. It may lead to premature treatment termination (up to 14.2% in one study),^15^ therapeutic failure, reinfection, antimicrobial resistance, and hospital readmission. Consequently, it increases the cost of therapy and reduces patient quality of life. In the USA, the cost of non-adherence in the year 2020 was approximately $300 billion.^168^

In addition to non-adherence, patients might completely reject enrolment in an OPAT programme. Firstly, role ambiguity is common among patients and caregivers in home-based OPAT programmes. Because of this, the performance of medical tasks after hospital discharge is challenging for caregivers and patients, resulting in OPAT rejection.^169^ Secondly, hazards from the physical home environment, namely bathing, caring for animals or pets, extremes in temperature, household clutter, indoor soil and food exposures, and mobility issues, hinder the safe performance of OPAT tasks.^170^ Thirdly, patients who have no social and/or financial support have reduced OPAT feasibility and patient acceptance.^171^

Beyond the aforementioned factors, OPAT enrolment may be restricted by insurance policies and home infusion companies. For instance, in the USA, Medicare does not cover sOPAT, leading to potential out-of-pocket costs for home infusion services and medication supplies for patients.^42^ Consequently, some Medicare patients may refuse home infusion services and opt to receive OPAT care at infusion centres or at an SNF to avoid out-of-pocket costs.^26,172^ Additionally, home infusion providers might reject services due to safety concerns, service area limitations, or lack of agreement with the patient’s insurance provider.^172^ Therefore, to enhance patient adherence and ensure successful completion of OPAT treatment, strategies such as proper follow-up and monitoring, including the use of telehealth as a remote monitoring tool, education about consequences of non-adherence, addressing home environment barriers, and negotiation with insurers for coverage may be considered.^13,149^

### Antimicrobial instabilities in OPAT practice

Antimicrobials should demonstrate chemical, physical and microbiological stabilities at the designated storage temperature and ‘in-use’ solution temperature during use in OPAT settings.^173^ Albeit limited, degradation of antimicrobials may occur to some extent at their selected storage conditions (usually refrigeration or at room temperature for stable antimicrobials). In addition, those antimicrobials administered via CI over 24 h are exposed to high in-use temperatures (solution temperatures of up to 32 ± 1°C), which risks degradation of thermolabile antimicrobials such as the β-lactams.^138^ Instability and subsequent degradation affect target concentration attainment and cause inadequate exposure to the pathogens, which increases the opportunity for antimicrobial resistance. Therefore, sequentially generated stability data are crucial to reflect OPAT infusion conditions because the solution may be stored for a long time, for example, a week in a refrigerator, prior to exposure to the 24 h run-out period at a higher temperature.^174^ Additional factors that may affect stability of antimicrobials in OPAT include the different diluents and additives used, the concentration within infusers, and the composition of infusion devices such as elastomeric infusion pumps.^144,175,176^ The acceptable limit of degradation is generally not more than 10% (or 5% in the UK),^138^ though some antimicrobials fail to meet these stability requirements (Table 4).

**Table 4.** Summary of chemical stability for some antimicrobials that are not suitable for 24 h CI in OPAT settings

Antimicrobial	Type of infusion device	Tested C, mg/mL	Diluent(s) used	Laboratory conditions (time and temperature)	Chemical stability status	References
Amoxicillin	Unspecified	50, 125, 250	SWFI	12 h at 25 °C	Not stable	^177^
	Baxter Infusor	25–83.3	NS	1 d at 4°C; 24 h at 25°C		^178^
	Baxter Infusor	50	SWFI	24 h at 25 ± 1°C		
Ampicillin	Not specified	50	RLS	10 d at 4°C; 24 h at 25°C; 24 h at 31.1°C	Not stable	^177^
	Baxter Folfusor	50	RA, NS, D5W, DES	24 h at 25°C	Not stable	^178^
	Baxter LV10	50	NS, RA, D5W, DES	24 h at 31.1 °C	Not stable	^179^
Cefepime	Baxter Infusor	6–50	NS	24 h at 35 ± 1°C	Not stable	^180^
	Unspecified	20	NS	33°C	Stable only for up to 12 h	^179^
Ceftaroline	Baxter LV10	6	NS	24 h at 35°C	Stable only for up to 12 h	^181^
			D5W	24 h at 35°C	Stable only for up to 6 h	
			NS, D5W	24 h at 30°C	Stable only for up to 12 h	
Ceftazidime	FOLFusor, Baxter and Easypump, B. Braun	12 and 25	NS	48 h at 2–8°C; then 12 h at 32°C	Stable for up to 12 h	^182^
Ceftolozane/tazobactam	Baxter Folfusor, B. Braun Easypump	5 and 20	NS	8 d at 2–8°C, plus 24 h at 32°C	Stable only for up to 12 h	^183^
Cefsulodin	Baxter Infusor	6–50	NS	24 h at 35 ± 1°C	Not stable	^178^
Doripenem	Baxter Folfusor	12.5	RA, DES, D5W	24 h at 25°C	Not stable	^178^
	Baxter LV10	12.5	ARS, DES, D5W	24 h at 31.1 °C	Not stable	^39^
Flucloxacillin	Unspecified	33	NS	24 h at 30.9 °C	Not stable	^177^
Meropenem	Baxter Folfusor	12.5	RA, NS, D5W, DES	24 h at 25°C	Not stable	^178^
		25	NS (buffered with 32% HCl)	24 h at 22.5°C	Not stable	
	Unspecified	6.25 and 25	NS (buffered with different conc. of citrate and phosphate)	24 h at 32 °C	Not stable	^184^
	Baxter LV10	12.5	NS, DES	31.1°C	5–6 h stability	^39^
Pen G potassium	Baxter Folfusor	100 U/mL	NS, D5W, DES	24 h at 25°C	Not stable	^185^
Piperacillin/tazobactam	Baxter Infusor	90/11.3	NS	24 h at 35 ± 1°C	Not stable	^178^
	Baxter Folfusor, B. Braun Easypump	50/6.25, 50/6.2 and 90/11.25	NS	24 h at 35 ± 1°C		
Temocillin	Easypump II and Dosi-Fusor	25/12, 50/3 and 25	0.3% citrate buffer at pH 7	14 d at 5°C ± 3°C, 24 h at 32°C	Stable only for up to 12 h	^186^

ARS, acetate Ringer solution; C, concentration; CI, continuous infusion; D5W, dextrose 5% in water; DES, dextrose-electrolyte solution; NS, normal saline; OPAT, outpatient parenteral antimicrobial therapy; RA, Ringer acetate; RLS, Ringer lactate solution; SWFI, sterile water for injection.

β-Lactam antibiotics are the most studied class of OPAT antimicrobials, given concerns about their stability. A systematic review by Perks *et al*.^174^ summarized a large volume of data from β-lactam stability studies. Accordingly, most of these studies lack regulatory compliance for OPAT use, revealing insufficient stability data in warmer climates (around 34°C or above). The most specific guidance in this regard is outlined in the UK’s National Health Service's (NHS) standard protocol for deriving and assessing stability, the so called Yellow Cover Document (YCD).^138^ The YCD extended infusion stability acceptance criteria are key requisites in the clinical governance and quality assurance of OPAT services.^187^ The YCD states a tolerance limit of 95%–105% for active pharmaceutical ingredient (API) in manufactured products unless a British Pharmacopoeia monograph exists to state the limit.^179^ In this regard, sequential stability studies that comply with this have been very limited.^174^ However, YCD-compliant stability studies have been increasing lately, with published reports available for buffered flucloxacillin,^188^ buffered piperacillin/tazobactam,^183^ ceftolozane/tazobactam,^183^ meropenem,^184^ ceftazidime,^182^ temocillin^186^ and cefazolin.^187^

The stability of 0.3% w/v citrate-buffered saline solutions of both flucloxacillin and piperacillin/tazobactam in two different infusers was significantly improved and complied with YCD standards following storage at 2–8°C for up to 2 weeks and for 24 h CI at 32°C, providing further opportunity to facilitate patient early discharge to the OPAT models of care. Another β-lactam stability study reported that saline and 5% dextrose solutions of piperacillin/tazobactam, cefazolin and cefmetazole remain greater than 90% of their zero-time concentration for 24 h CI at 31.1 °C following storage for 10 days at 4 °C. ^39^ Jenkins *et al*.^187^ extended the work by Perks *et al*.^174^ and provided data to support the use of cefazolin in elastomeric devices that meet YCD criteria.

Unfortunately, this is not the case for some other key β-lactams. According to Jamieson *et al.*,^184^ solutions of ceftolozane/tazobactam (5 mg/mL and 20 mg/mL), ceftazidime (12 mg/mL and 25 mg/mL) and meropenem (6.25 mg/mL and 25 mg/mL) did not meet the YCD requirements for 24 h CI considering a ≤5% degradation limit. However, where the degradation profile is clear and the risk of toxicity from any degradant is considered negligible, 24 h CI may be possible with degradation rates >5% as long as the drug meets the required clinical activity (key pharmacokinetic/pharmacodynamic targets), as recently described for ceftolozane/tazobactam.^189^ Ceftazidime, with the 90%–100% API limit and the 0.5% (w/w) pyridine limit, is also stable only for a 12 h CI at 32°C after refrigeration for up to 48 h. Similar findings were reported for temocillin evaluated in 0.3% citrate buffer at pH 7. For meropenem, given the YCD 95% minimum content limit, the acceptable infusion period is only up to 6 h. Patients may benefit from co-administration of oral probenecid as it lowers meropenem renal excretion while increasing its concentration and half-life.

### Challenges related to OPAT structure and administration

A well-designed structure, as well as skilled, communicative and committed team members, are key components for favourable outcomes during OPAT practice.^190^ In addition, dedicated leadership and funding support are crucial for a successful OPAT programme. However, it is difficult to have all these simultaneously, and limitations are apparent in different practice settings. Lack of leaders’ awareness of OPAT values, insufficient IT support, and limited knowledge among interdisciplinary members about medical devices are some of the common limitations.^191^ Financial constraints and, consequently, a lack of incentives are also usual challenges.^28,192^ In some jurisdictions, such as in Belgium, the parenteral antimicrobial reimbursement approval certificate was not uniform and even delayed.^192^ According to reports, there was also poor communication among OPAT providers, affecting the logistics, and this led to low availability of medicines in the community pharmacies.^191,192^ Such structural, managerial and interdisciplinary barriers remain among the challenging aspects of OPAT practice across the globe.

### Unplanned hospital readmission and its predictors

Outpatients receiving antimicrobials specifically in a home-based model of care are at risk of adverse complications associated with antimicrobials or the catheters used.^193^ In addition, vulnerable population groups are at higher risk for drug-related adverse events, line-associated complications, and non-adherence that leads to hospital readmission.^50^ Hospital readmission rates are variable between regions, with reported rates ranging from 6% to 26%.^19,166,194^ However, among vulnerable populations, such as PWID, a higher hospital readmission rate (41%) has been reported.^195^

Unplanned hospital readmission is a burden to the patient and the healthcare system. Readmission increases patient stress, healthcare costs, risk of additional infection and subsequent patient mortality rate.^196^ Consumption of more beds and resources restricts the capacity of the healthcare system to provide the required services for severely ill patients.^161^

**Figure 3.**
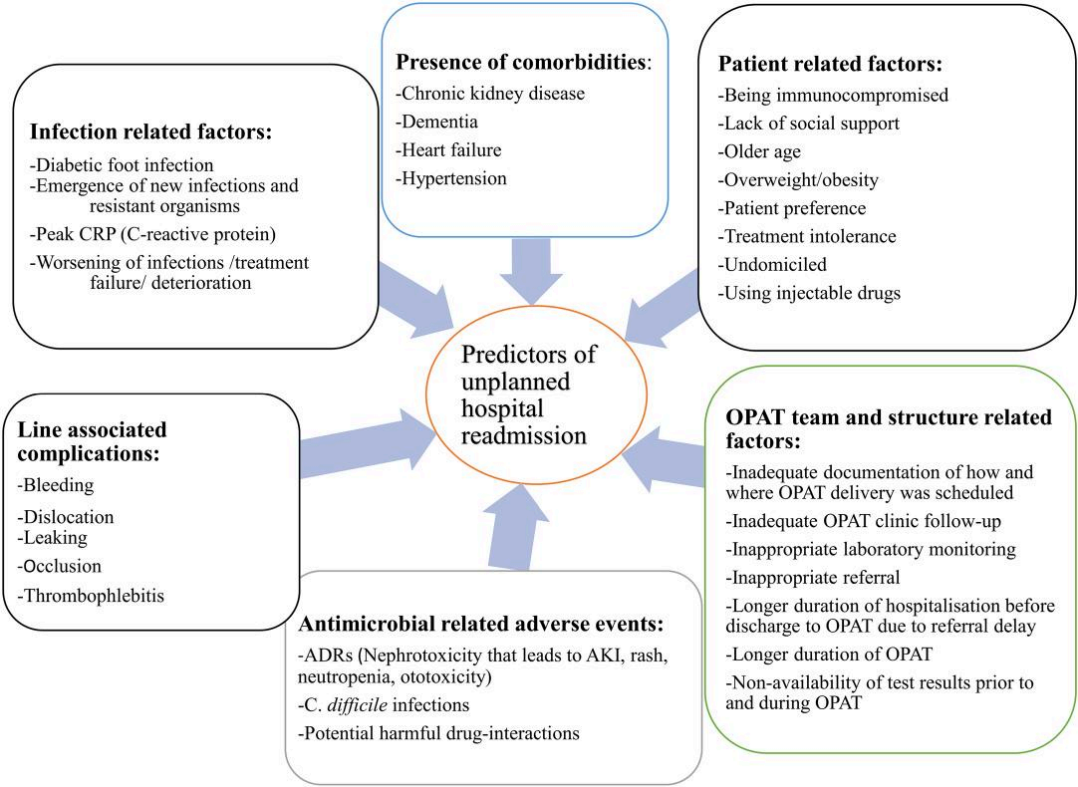
Predictors of unplanned hospital readmissions among OPAT patients. ADR, adverse drug reaction; AKI, acute kidney injury; OPAT, outpatient parenteral therapy. This figure appears in colour in the online version of *JAC* and in black and white in the print version of *JAC*.

Several previous studies reported different predictors for hospital readmissions (Figure 3). Complications associated with antimicrobials and catheters, patient-related factors, factors related to the OPAT team and structure, infection-related factors and presence of comorbidities such as cardiovascular and CNS disease were cited predictors of unplanned hospital readmission due to their impact on patient health, treatment effectiveness and post-discharge complications.^118,166,193,194,197–199^

## Conclusions

The practice of OPAT continues to grow in middle- to high-income countries because of its multiple benefits. As a novel treatment alternative to inpatient hospital care, it has proven safety, efficacy and cost-effectiveness. With predetermined eligibility criteria, many patient populations can be considered for such a programme, although close follow-up by multidisciplinary team members, including laboratory and clinical assessments, is necessary following its initiation. Several infections requiring prolonged IV antimicrobial therapy are successfully managed through OPAT programmes. However, multiple challenges are counteracting its expansion. An AMS programme is essential to optimize the use of OPAT and minimize some of the associated challenges faced in clinical practice.
